# Professional narratives on continued training in the laboral landscape of social education

**DOI:** 10.3389/fsoc.2024.1419946

**Published:** 2024-07-18

**Authors:** Margarita Campillo Díaz, Amalia Ayala de la Peña, José Santiago Álvarez Muñoz, Ma Ángeles Hernández Prados

**Affiliations:** Department Theory and History of Education, University of Murcia, Murcia, Spain

**Keywords:** formative needs, profession, social education, continuing training, professional narratives

## Abstract

**Introduction:**

Labor consolidation is not a sufficient criterion to abandon the need for the qualification and requalification of professionals, especially in the field of education, which must respond to the uncertainties of society in the light of trends and advances incorporated in pedagogical research.

**Methods:**

The present qualitative study analyzes the training demands of social education professionals from their own perspective, using their own stories in a semi-structured interview format conducted with), key informants from governmental and non-governmental organizations in southeastern Spain, specifically from the autonomous community of the region of Murcia.

**Results:**

According to the results, the most frequent and significant formative limitations are those referred to in the field of social policies, legislative training, administrative processing, specialized work in specific sectors or collectives, and mediation. Similarly, the results reveal fashion themes (mental health, gender) and reiteration of non-exclusive conditioners of social education. These are extrapolable to other areas, such as the increasing bureaucracy and complexity in the proceedings as common places in the reflection of professionals with their own initiative and commitment to their own updating that is associated with a reflective criticism of their own professionalization.

**Discussion:**

The diverse range of responses and subjects, as indicated by the numerous descriptors needed for the categorization and their respective percentages, leads us to conclude that ongoing professional training does not encourage excessive specialization. Instead, it necessitates offering a broad range of training adjusted to the versatility of situations.

## 1 Introduction

The environments and situations that require socio-educational intervention are in themselves complex and suggestive cresols of the limitations and challenges facing our societies (Miguel et al., 2022). Similarly, the increase and complexity of educational environments and participants and the evolution of social reality generate a favorable environment for consolidating initiatives from social education (García-Vita et al., 2020). They are an opportunity to think about the societies we are part of, to which we want to contribute, and to which we commit our professional exercise, care, and time. The modern age has experienced rapid technological advances, continuous political and social changes, migratory movements, unprecedented refugee situations, and even a global pandemic. All this contributes to a volatile environment reflected in all aspects of life, including education. On the other hand, this situation of uncertainty also includes many other almost chronicled constants of a structural nature or the perpetuation of unsustainable situations. It poses various educational challenges, which open up new opportunities to rethink how education is approached in the 21st century.

The work paths of social education practitioners represent irreplaceable baggage in a desirable and necessary approach to continuing training. We are in a framework of socio-educational intervention, in which frequent exclusion provides the population served by social educators with diverse, specific, and changing needs. It is precisely responses that are sensitive to this social reality that can contribute to improved or, in any case, better-adapted attention to the necessary responses and that, wheelieing the necessary flexibility, escape improvisation (Esteban et al., 2017; García-Vita et al., 2020). Accordingly, the practice of social education is complex. It requires flexibility to serve vulnerable individuals and groups in diverse contexts, combining theoretical and practical understanding and focusing on integrating and recognizing the rights of the marginalized, both within and outside the walls of any institution, including the school (Azevedo, 2019; Da Silva et al., 2021).

The formative demands that have been considered by the social educators in practice, which have been formulated, demanded, and/or achieved, may imply the awareness of the adequacy or inadequacy of their responses to some of these uncertainties of our time. Identifying shortcomings is always a good first step in finding ways to solve or improve. Within the framework of the professional development of social educators effectively involved in the dynamics of continuing education, the approach of the desideratum, expressed for the continuing training of teachers of different fields and levels, “not as a particular and voluntary option but as a question of ethics” (Escudero Muñoz, 2020, p. 110). A commitment to professional development is not given in a vacuum. It is not the ultimate responsibility of the professional alone; it must incorporate various co-responsibilities and synergies that contribute to creating local and systemic environments that are favorable to good continuous training (Escudero Muñoz, 2020). This starting point, which necessarily includes the systemic reference, already finds particular resistance in the regulated educational system. It becomes more complex, given the different areas of action of the social education professional, the specificity of the situations and populations served, the problems concerned, and the diversity and variety of the frameworks of action in which the work of the social education professional is performed.

Furthermore, characterizing perceived needs for continuing training is not restricted to the new scenarios or changing situations mentioned at the outset. The formative shortcomings of a qualitative inquiry approach that listens to the voices of social educators in practice (Bretones et al., 2014; Eslava-Suanes et al., 2018a; Hernández-Prados and Ayala de la Peña, 2021), where the experience of initial and continued training on the one hand, and the professional exercise on the other, activates the capacity for a critical and contextualized reflection. This will be the right starting point for constructing and deconstructing continuing training processes that contribute to understanding existing proposals in this field and their challenges and limitations.

### 1.1 What social education for what society?

Social education is a professional practice that operates in specific societies with particular situations, resulting, among other issues, from the prevailing ideology and the role of the institutions and the market in the lives of each citizen. The social educator is an education professional working closely with people. Social education, therefore, should be focused more on prevention and oriented toward the intellectual development and formation of citizens and not just deficit-oriented (Ponce de León and Castro, 2014; Kraus and Hoferková, 2016). In this regard, social education professionals must know the problems that arise in the social fabric where they are placed, characterized by uncertainty, change, technology, and the current.

Uncertainty appears as a central axis of thinking, characterized by globalization, greening, digitization, and individualization in the workplace. Regarding the question Ulrich Beck pointed out in 1998 in his book, *The Risk Society*, we can ask ourselves the following today: Why is society put at risk if technological advances provide us with greater security than ever and greater control over all areas of our lives? Because we exist, we have a common horizon marked by fear, in which the risks appear as threats because of the past. At the same time, they are projective because they ask us what we can do today in the face of the reality that they present, such as current wars, conflicts of devastating consequences, the threat of nuclear war, food pollution, climate change, etc. There are indeed problems that affect a few, but others affect every living being who struggles for an uncertain future (Beck, 1998).

The interest in change, which defines today's society and calls for social education, comes from some questions we must answer: in what direction is this change and progress oriented? Who benefits? And what do social education professionals do to respond to this? To reflect and respond to these issues, we resort to some ideas that can be identified in our society and determine how we settle in it. For instance, the idea “who doesn't change stops; he who does not progress, returns” is a daily vision that becomes a prescription and that, at times, encourages submission and forces us to be in continuous growth and movement to try to satisfy it. We need to evolve, change, and improve. In schools, institutes, universities, and other institutions of knowledge, including the media, this value tends to be understood as natural as an identity of the human being. All these institutions and news agencies practice the “chantage against delay” (Brunet, 1996): you do not have to stop; you have to buy without limits, and you must bet on the new—new computers, new cars, mobile, fashion, instruments, etc. everything that responds quickly to what is offered to the citizens on a daily basis.

The question here, and we focus on the second question, is who benefits from that vision of change, as it sometimes produces irreversible, undesirable effects that question the legitimacy of progress as a value to follow. Sometimes, this particular interpretation of progress has triggered and can trigger ethical decline. When progress, change, and material growth occur without limit, it can result in excessively consumerist and mercantile societies that, without exercise or democratic distribution, lead to exclusion, marginalization, and crime (Jauregui, 1997), as a consequence of unequal and discriminatory policies.

In this case, what is the response from social education? This is the third question. In its ethical component, social education has collaboration, consensus, participation, and democratic asymmetric relations—one of its most relevant weapons in enhancing values such as cooperation, joint work, distributive justice, solidarity, and professional encouragement.

Related to change, there is another feature of the surrounding social fabric, such as technology. Technology, together with speed, has become the major reference of postmodernity. We live in a hurry, think rapidly, and experience the vertigo of speed. This is not intended to undermine the benefits of technology as an instrument but to warn of the consequences of technology or change only as an end. The value of technology and its false association with speed entails counter-values that harm human nature, leading to anxieties, mental and physical diseases, disruptions resulting from continued failures in trying to compete based on economic and social success, etc. (Accoce and Rentchnick, 1996). At the same time, at the social level, it triggers economic and social inequalities between countries and communities that feel and live at a different pace (Sáez and Campillo, 2014). Virilio (1995) suggests that the invention of a real-time perspective for the 21st century, in which cyberspace allows not only to see and hear but also contact, is transforming human perception and interactions. This transformation is distorting reality and generating a disconnection with the other and with the environment, which could potentially trigger a deep social and democratic crisis. As UNESCO warned in 1985, the uncontrolled development of such technologies and the content they transmit could aggravate cultural and economic inequalities and be detrimental to local, regional, or national cultural expression. Many years later, echoing those words, it would be necessary to ask whether the technology has contributed to exclusion, racism, and marginalization in the so-called “developed civilizations.” Technology and technology are a reality and key at the moment, but we have to be careful and not give them the value of law just because that is what the times want.

Finally, a lack of questioning of the “new.” “New” has made the present the supreme value that evokes a requirement, such as to adapt to the times, because the present time decides what is important in each moment (Wajcman, 2020). This obsession with the present has elevated privatization, consumption, speed, efficiency, and being efficient in what we do above purposes and people. Even advertising, social networks, and communication remain mere information, which requires blind adherence and absolute characterization (Brunet, 1996). For instance, talking about the classics in Literature or Music is out of play. Not being up to date with the new characters that dictate life forms means not being up-to-date and feeling marginalized from what is happening. We live in a society of entertainment or spectacle if we use Debord's terminology (Debord et al., 1990; Morelock and Narita, 2021). Educators feel that pressure in their educational work every day. Consequently, at least three alternatives are proposed: reaffirm the role of education as a social regulator, reclaiming the traditional role of education; adapt by trying to be fun; or resist education by attempting to combine the virtualities offered by the media as resources with the empowerment of learning that reinforces critical thinking that sometimes disappears with the values of the present era. The critical aspect, necessarily constructive, is insurmountably engaged in social education interventions.

### 1.2 Current training needs of social educators

The characteristics of the social fabric described above have consequences in citizenship that have interpellated the professionals of social education, which translates into training needs for their professional performance or mastery over a set of expertise to address certain types of problems. In reality, the profession of social education has materialized in each situation and context in a different way and with different intensity (Garcia and Sáez, 2021; Sáez and Campillo, 2023).

Starting from the model of professionalization of Sáez (2004), we can say that there is a stage of pre-professionalization, where the responsible actor is the university with its resources (accreditation, research, and training). The next stage is a stage of professionalization or de-professionalization according to how to influence different actors (university, state, labor market, professionals, and users) (Sáez, 2004) in the professional development of this educational practice, qualification, and profession of social education.

If we focus on the stage of pre-professionalization, the training given at the university to professionals to develop scientific knowledge and form critical citizenship requires several conditions for its success (Sáez and Campillo, 2014). Each teacher responsible for that training should consider the kind of professional that needs to be trained, study the professional practice to align academic logic with practice, and know the functions and competencies required in professional practice.

At the same time, after the professional has entered the labor market, they are at risk of de-professionalization, especially when political, economic, social, and cultural labor contingencies make it difficult to justify their professional identity. They find it difficult to have limited jurisdiction over the services they provide and to justify their legitimacy as a profession.

The characteristics of the social fabric that surrounds us and the different factors that determine the professionalization or de-professionalization of social educators lead us to investigate their training needs (Eslava-Suanes et al., 2018a) in the pre-professional stage (university) and continued training that facilitates their process of professionalization in a dynamic reality that demands to meet new needs that are generated before different problem situations around the subjects. Accordingly, we examined the following research problem: What perception do social educators in practice have about continuing training? Specifically, we set out the following objectives for the study:

1. Analyze the value that social educators attach to continuing training; in particular, identify whether or not they perceive it necessary to improve and update their professional practice.
2. Identify the training that is considered a priority in developing the roles demanded in social educator practice.
3. Identify the main themes that, according to the perception of active social educators, should be the focus of training in their pre-professionalization and professionalization phases.

## 2 Method

### 2.1 Design of investigation

A mixed methodological design was used to deepen the understanding of the training demands of social educators in their work context. This design was adopted to abandon dichotomous research models and opt for the confluence and complementarity of quantitative and qualitative methods. The mixed approach allowed information to be collected from semi-structured interviews framed in a hermeneutic paradigm (Flick, 2007). It was approached from the sequential exploratory design proposed by Ortega-Sánchez and Heras-Sevilla (2021) for the analysis of the data obtained through the following phases: definition of evaluative categories, identification and coding of text segments relevant to the evaluative category in question, compilation of the text segments coded with the same code, and quantification and content analysis. For the analysis of the content, the following guidelines established by Lindseth and Norberg (2004) were taken into consideration: naive reading, repeated reading to grasp the idea and meaning of the text; structural analysis, division of the text into meaning units for better organization and meaning; and broad comprehension, critical reading from the identification of the text as a whole that is interpreted from the context.

The qualitative study model of this study was developed from a narrative analysis that, unlike content or discourse analysis, studies the subjective experience of the subjects of perceiving a specific phenomenon. The interest of the research resided in the educational encounter generated in the narrative dialogue (Bolívar Botía, 2002; Dávila and Argnani, 2020) and making visible the voice of practicing professionals in their work environment regarding functions, professional competencies, and training needs. Direct, genuine, and personal contact was sought through interviews, which required a labor-intensive strategy of access to practicing social educators (Kvale, 2011). The narrative analysis was framed in different typologies according to the author. On the one hand, following Riessman's (2008) classification, this study can be seen as a thematic analysis since it paid special attention to the content of the narrative; on the other hand, from Lieblich and Tuval's (1998) perspective, it can be viewed as a categorical content approach since it analyzes the entire content from quantification by categories.

### 2.2 Sample

The sample was obtained using a non-probabilistic convenience sampling technology, using the snowball technology to obtain the maximum sample in a short period of time. This was based on contacting some potential participants who met the criteria so that they could disseminate the information to other potential participants, and the process was repeated. This sampling has several advantages, such as access to difficult populations, easy identification of the target sample, and less expensive. However, it also has many disadvantages, such as a representativeness bias, dependence on external agents, and limitations in generalizing results. In this process, the inclusion criteria were to be a graduate in social education and to work as a social educator in a center in the Autonomous Community of the Region of Murcia. Exclusion criteria included not practicing, having a different degree, or carrying out professional work outside the territorial scope of the study.

In this way, the research had a final sample of 75 social educators (54 women and 25 men). According to their graduation year, 18.4% graduated between 2006 and 2010, 20% between 2011 and 2015, 30.1% between 2016 and 2020, and 31.5% over the past 3 years. Concerning work experience, 56.3% had worked for <5 years, 23.3% had experience of 5–10 years, and 20.4% had more than 15 years working as a social educator. Finally, 24.5% worked in public administrations and institutions, while 75.5% worked in private institutions such as NGOs, foundations, companies, and associations.

### 2.3 Procedures of data collection

The interrogation technology was applied to obtain information using an open-structured interview that included a series of open-ended questions. For its preparation, a panel of experts was created with four university professors specialized in the professionalization of social education, thereby guaranteeing the validity of content. Based on their feedback, the draft version of the instrument was prepared and subsequently validated by the members of the research team specializing in social education at the University of Murcia. The final version of the questionnaire, titled “Interview with social education professionals: Continuing training needs of social education practitioners,” consisted of 13 open-ended questions. However, in the present survey, only six were chosen, specifically those related to the analysis of continuing education needs. These were:

1. What aspects do you consider essential in the vocational training of social education that have not been received previously in the graduate training or which you consider necessary to strengthen?
2. Have you had the need to receive follow-up training during these years as a professional? Yes/No In what aspects or topics?
3. Have you completed any training courses after completing the degree? Yes/No Which ones?
4. Is it difficult to find continued training in any particular area? Yes/No In what ways?
5. What training offer do you have at your disposal?
6. Identify at least three topics that are essential for the follow-up training of social education professionals.

### 2.4 Procedure of investigation

After designing the questionnaire, the process of application and data collection began. To this end, contact was established (via e-mail) with associations, entities, administrations, and organizations that hosted students with a Degree in Social Education for internship. The e-mail requested the collaboration of the social educators in completing the interview, provided the link (Google Form) to access and complete the interview, and also included an information sheet on the research. A period of 15 days was provided to complete the interview. A week later, a reminder was sent on the expiration of the deadline. To increase participation, telephone contact was established with the managers of those centers with a low rate of participation. Finally, all participants who voluntarily answered the interview were sent a thank you note. This entire research procedure was carried out from September to December 2023.

The entire research procedure considered ethical parameters as one of its guiding principles. To begin with, the design of the research procedure was assessed by a team of educational researchers with extensive experience in the framework, paying special attention to compliance with research ethics. In addition, the completion process was carried out in accordance with the Helsinki Declaration, which is related to informed consent, anonymization of the participant, and data coding, as well as respecting the participant's identity at all times. The APA regulations were also respected, including compliance with the privacy and data protection policy (standard 4.2). Participants were also informed that they could leave the research any time they wished (standard 8.2).

### 2.5 Data analysis

First, after grouping all the data from the completed interviews, we began with the analysis of the narratives following the steps established by Hernández-Sampieri (2004):

- Definition of the universe: establish what reality will be the object of study, in this case, the responses of the social educators to the questions posed by the semi-structured interview.
- Definition of units of analysis: which variables will be measured for information collection. In this case, it focuses only on the content.
- Definition of the categories of analysis: Clarification of which levels are established to categorize or classify the units of analysis. In this case, we start from a previously pre-established theoretical model, specifically that of Álvarez (2017), which determines the areas of work of the social educator from a series of perspectives: type of intervention, group, context of intervention, and dimensions of the person, being able to associate the training requirement to this subdivision. This theoretical model is applied to specific questions of analysis 1, 2, 3, and 6 since it is closely linked to the training demands, narrowing down categorization themes. However, for specific objectives 4 and 5, limitations and agents, respectively, categorization is done through triangulation since there is no previous theoretical model.
- Selection of coders. The content was assessed by three university experts with experiential and scientific backgrounds in social education.
- Elaboration of coding sheets: Template for the grouping of text segments by users and, in turn, for the establishment of frequencies of the units of analysis.
- Triangulation of the codes. The moment in which the coders present their analyses and reach a common consensus to establish the final analysis.
- Coding. Establishing the repetition frequencies of the categories, i.e., the number of units that fall into each category.
- Gap of information.

A digital transcription of the interviews was carried out to conduct the analysis correctly. After that, the answers to the six questions analyzed were categorized; in this procedure, through the triangulation of the data, three researchers determined the categories and their frequency so that the subjectivity bias could be eliminated. After delimiting the categories and specifying the frequency with which each is present, all the comments associated with selecting that category were displayed in a log sheet, indicating which participant was the author of the said comment, for example, P23, being participant number 23.

## 3 Results

The results of the qualitative analysis and quantification by categories of the interviews analyzed are presented in this section. The tables show the frequencies and percentages of each of the questions included in the open-ended interview. In this research, given the magnitude of the instrument and the amount of information it contained, an exhaustive analysis of the initial and continuing education block was made. Therefore, the results section is organized according to the following order of appearance: Training aspects to be reinforced in the initial training of social educators; Continuing and ongoing training needs identified at the beginning of professional practice; Continuing training received by social educators; Limits on access to continuing training for social educators; Agents promoting continuing training for social educators; and, finally, essential themes of continuing training for social educators according to their own criteria. Sections 3.1, 3.2, 3.3, and 3.6 are approached from the analysis of the theoretical training model of the social educator proposed by Álvarez (2017), contemplating three major dimensions and their categories, namely, type of intervention, context of intervention, and dimensions of the person. In Sections 3.4 and 3.5, the information categorized from triangulation is presented. Since there is no previous theoretical model in this regard, it is presented as is.

### 3.1 Introspective evaluation of the initial training of the social educator

From a reflective and critical vision of the lived reality, social education professionals should identify the aspects of improvement of the training received during their university degree. In general, the data reveal that 92% of those surveyed consider that the initial training received requires some improvement. In the best of cases, they suggest a complementary deepening in some aspects that need to be reinforced. Only 8% did not answer the question or stated that no aspect required improvement.

The categorization of micro-narratives based on the theoretical model of Álvarez (2017) revealed that 39.77% appreciated a higher need for previous academic training in intervention practices; 35.21% focused on the contexts of intervention; and, finally, 24.99% was related to the dimensions of the person. As seen in Table 1, the categories of the three dimensions are provided in detail.

**Table 1:** Essential aspects of the professionalization of the social educator have not been addressed or need to be reinforced in the university degree.

**Dimension**	**Category**	***N***	**%**
Type of intervention (*N* = 35; 39.77%)	Intervention processes	18	20.44%
	Mediation	8	9.08%
	Counseling	7	7.95%
	Management	2	2.27%
Contexts of intervention (*N* = 31; 35.21%)	Specialization	11	12.49%
	Immigration centers	9	10.22%
	Dependency centers	7	7.95%
	Centers for minors	4	4.55%
Person dimensions (*N* = 22; 24.99%)	Health	10	11.36%
	Social relations	6	6.81%
	Family	3	3.40%
	Leisure and free time	2	2.27%
Work	1	1.13%

The main demand identified in the intervention is the need for more practical training that provides tools, technologies, and strategies for direct intervention with the user, regardless of the group (20.44%). The social educators themselves complained about the overload of theory in their training, which is difficult to translate to work practice: “The degree consists of much theory and much theory... we do not have any type of reference, tools, training or strategies for it (P41).” Similarly, participants also expressed the need for a systematization of the theory so that it can be exercised effectively in the praxis of the social educator: “to know the steps or tools of the processes in the practice itself (P39).” Moreover, the emphasis was not on replicating the subjects but on the need to incorporate external practices within the curricula: “Many people study social education without having the necessary vocation, and when they enter the working world, they are not efficient due to frustration. If at the university they had the opportunity to do internships in all courses, they could find out if the degree really meets their expectations or not (P65)”. Respondents emphasized the importance of training and checking the viability of the person for this professional work.

On the type of intervention, although with a lower rank, mediation stood out as another of the previous unmet training demands (9.08%). The interviewees considered that it should be approached from a specific subject (P34; P39) that affects contents such as negotiation technologies (P11) or procedures for action in crises (P50). They also stated the need for knowledge in legislative aspects for the correct counseling of the users with whom they work (7.95%): “I missed more legislative training (P56)” to have acquired the frameworks of action of social policies (P73). Management (2.27%) was the least prominent option among the types of intervention, as stated by two interviewees in response to the need to know the documentation for the regulation of entities (P45; P73). Therefore, it can be concluded that there is a greater demand for practical training related to contact with the user from the processes of intervention and the practice itself in a generic way and specifically pointed out mediation. Meanwhile, with a lower representation, it is clear that there is little knowledge of the management and counseling frameworks (policies, legislation, procedures, etc.).

The context of intervention was the second most present dimension. However, 12.49% of the interviewees stated that there was a lack of specialized training, i.e., greater specialization of the various groups on which the intervention was focused. More specifically, most of the contributions are limited to indicating the need to theoretically or practically deepen with the different profiles or groups (P40, P41, P43, P45, P70). Yet, only one of them raised the alternative of structural reform of the degree, proposing specialization itineraries depending on the areas or groups: “Social Education covers many areas. It would be good if there were a specialization within our diploma or degree (P74)”. Apart from generic approaches, specific contexts were also discussed in depth. One of the most frequently mentioned was immigration centers (10.22%), highlighting the obligation to know the policies (P19), documentation (P60), or naturalization processes (P60) to offer a better intervention for their reintegration. The vulnerability of immigrant women was also mentioned as an increasingly demanding field of work but with less training in the field of university degrees (P51; P61; P66). Other groups mentioned were drug addicts (P43; P22) who attended from the centers of dependence, with knowledge of the pathologies, their consequences, and strategies for their abstinence. Lastly, 4.55% mentioned centers for minors and the training deficit concerning educational attention to this particular type of public and its peculiarities (P14; P25).

The least mentioned dimension was the dimensions of people. Within it, the area of health development was the most demanded (11.36%). Mental health was recognized as an emerging field of action with urgent action from several professions; one of the most recently considered is that of the social educator (P23; P43). The level of social relations (6.81%) is also worth mentioning, placing the social educator as responsible for supporting the user so that they can form healthy and productive social relations (P34; P56; P76). Finally, family (3.40%), leisure and free time (2.27%), and work (1.13%) were the dimensions of the person that were least quantified through the interviews.

### 3.2 Continuing and lifelong training needs identified at the beginning of the career

The following are the main continuing and ongoing training needs that social education professionals identified at the beginning of their professional practice. We organized the information into 14 categories within the three dimensions of analysis to determine their greater or lesser presence (see Table 2). In this regard, 85% of the social educators confirm that they have found training deficiencies, identifying as the most frequent: management, specialization, or the user's social relations, among others. According to the dimensions, in this case, the context of intervention prevails as a training need at the beginning of the professional career (41.22%), well-above the types of intervention (34.21%), or the dimensions of the person (24.56%).

**Table 2:** Essential aspects in the professionalization of social educators in which they have indicated limitations and requested further training.

**Dimension**	**Category**	***N***	***%***
Type of intervention (*N* = 39; 34.21%)	Management	15	13.15%
	Mediation	11	9.64%
	Intervention processes	7	6.14%
	Professionalization	6	5.26%
Contexts of intervention (*N* = 47; 41.22%)	Specialization	14	12.28%
	Dependency centers	11	9.64%
	Centers for minors	10	8.77%
	Women's centers	7	6.14%
	Immigration centers	5	4.38%
Person dimensions (*N* = 28; 24.56%)	Social relations	10	8.77%
	Health	9	7.89%
	Family	4	3.50%
	Leisure and free time	4	3.50%
	Work	1	0.87%

As in the previous question, concerning the contexts of intervention, the need for specialization is again expressed (12.28%) that emerges before the appearance of a first work activity with a specific group: “From my point of view, we must be in continuous training attending to the needs of the users with whom we count. (P39); Type of intervention with certain profiles (P54); knowing specific resources to attend to specific groups (P59),” declaring a series of shortcomings resulting from the absence of a specific itinerary, mandatory or optional, in the university degree (P12; P34; P67). Considering specific groups, the public with high dependency is the most indicated by the interviewees (9.64%), alleging the need for training in treatment and care (E45), knowledge of the law on dependency (P71), or the identification of biopathologies (P32). Minors are another of the contexts of intervention that require more training at the beginning of their professional career (8.77%), highlighting mental health, distinct from behavioral problems (P14; P56), eating disorders, or the risk of self-injurious behavior (P28). Women (6.14%) and immigrants (4.38%) were the least mentioned in the interviews.

Although the type of intervention is not the most mentioned dimension, it is the category that is most mentioned (13.15%); for instance, the one related to legislative and documentary bureaucracy, i.e., management. Intervention is identified from work in public institutions (P33; P45; P66), with special mention of social services and the documentation required to present projects and obtain funding (P19; P34; P71). Here, the importance of the legislative level is highlighted, given that, in the absence of jurists in socio-educational institutions, it is a competence that is increasingly required of social educators. As a previous training requirement, mediation and conflict management (14.63%) are also mentioned as emerging training needs after the university stage: “Yes, subject of contentions, conflict management (P9)” and “Conflict management in intervention (P49)”. Practical knowledge in intervention processes also stands out (6.14%), once again highlighting the deficiencies of the university degree in practical issues that impact the performance of the social educator profession: “The degree in social education needs to train students more in practice and in the interventions, they can carry out in relation to the different areas (P53)”. To conclude, in the type of intervention dimension, professionalization is the least commented on by the social educators interviewed (5.26%), a demand that is urgent in the face of labor intrusiveness (P55) and having a complete professional profile to achieve a better job situation (P40; P70).

As in the case of university education, the dimension of the person's spheres is also the least mentioned. Within it, on the one hand, social relations (8.77%) are mentioned given the emergence of social inclusion (P45) and the elimination of conflicts between users (E43), providing instruction to users on how they should relate to each other, and, on the other hand, health (7.89%), as a result of all the threats to users' health, whether mental (P65), physical (P21), or emotional (P33). Finally, mention is made of the need to attend to the context of the person beyond that of the institution or more formal education, demanding training to know how to act with the family (P23; P65) to have a more comprehensive intervention and from the exercise of leisure and free time (P11; P3), taking advantage of a more recreational and relaxed environment for the intervention.

### 3.3 Continuing training received by social educators

Table 3 shows the different important training the participating social educators have received after their university training, with 90% stating that they have undergone training divided into 12 categories that comprise a wide and varied training spectrum. In this case, the contexts of intervention (40.3%) are again the most commented on by the interviewees, followed by the dimensions of the person (34.21%) and the type of intervention (25.57%).

**Table 3:** Training received after a university degree.

**Dimension**	**Category**	***N***	***%***
Type of intervention (*N* = 33; 25.57%)	Mediation	18	13.95%
	Management	8	6.2%
	Insertion	7	5.42%
Contexts of intervention (*N* = 52; 40.3%)	Women's center	17	13.17%
	Centers for minors	13	10.07%
	Dependency centers	11	8.52%
	Immigration centers	11	8.52%
Person dimensions (*N* = 44; 34.21%)	Health	18	13.95%
	Work	9	6.97%
	Social relations	8	6.20%
	Leisure and free time	6	4.65%
	Family	3	2.32%

Within the context of intervention dimension, women is one area where social educators seek more continuing education (22.61%) due to the presence of women who are vulnerable due to mistreatment (P46), immigration (P45), or in the population of youth who are subject to sexual disorders (P71). The other population targeted by the training is youth (10.07%), given that several of the social educators state that a large part of the labor supply is in this field of action with radicalized minors, which is why a large number of them carry out their work in these contexts (P2; P23; P39). The last two groups, with 8.52% each, are immigration and disability. The former is mentioned because it is context-sensitive to various legislative, inclusive, labor, or sexist issues (P57; P41; P45). In the case of disability, the importance of training is stated to know the pathologies and, in this way, to carry out a better intervention (P16; P46; P75).

The category of the dimension of work with the user in which social educators in professional practice are most trained is health (13.95%), highlighting the importance of working on the mental health of their users to achieve integral development (P23; P45; P66). With a considerable difference, but still remarkable, training is also attended to learn how to provide job guidance to users (6.97%), highlighting this subject as training for employment (P56). Finally, social relationships (6.20%), leisure and free time (4.65%), and family (2.32%) are the training topics of the dimensions of the person that are least mentioned by the social educators in the interviews. They all agree on the need to work in these areas because they go beyond the institutional, emphasizing that the work of the social educator sometimes goes beyond the center where they work (P34; P45; P51; P43).

Finally, in the dimension of the type of intervention, mediation (13.95%) is the most mentioned, indicating that it is not only an initial training demand but also a continuous one. It is mentioned as a training field that is institutionalized and generalized within formal training in the professionalization of social educators and other professions. The suggestions include. “Course: conflict mediator” (P65), “Mediation families” (P10; P21; P23). “Specialist in Mediation” (P36), “socio-educational mediator, judicial expertise” (P33), “cycle in mediation” (P20), “Yes, a course on communicative mediation” (P42), and “mental health to adapt completely to my field of work” (P52). Management (6.2%) and Socio-educational inclusion (5.42%) are mentioned less frequently, suggesting their demand for knowledge of legislation and procedures and the need to educate the user and society for their inclusion, respectively.

### 3.4 Limits on access to continuing education for social educators

As can be seen in Table 4, the responses to the constraints are analyzed to identify continuing training. Thirty-three percent of educators identify restricted access to the training they wish to receive after graduation.

**Table 4:** Difficulties in the search for continuing training.

**Category**	***N***	**%**	**Category**	***N***	**%**
Lack of specific training	10	13.33%	Access	3	3.99%
Small variety	7	9.31%	Conciliation	2	2.66%
Economy	5	6.65%	Certification	2	2.66%

The main limitation is the difficulty in finding training that meets specific needs (13.33%). The difficulty in searching for the appropriate training is shared by more than one participant: “You have to go to highly specialized entities to find specific training. (P34)”; “By being so hidden for many years, the field of social things are missing many formations, yet they are necessary to be able to work with the collectives (P7)”. Others simply mention subjects about which they do not find the required training: “On Foreign Affairs (P29)”; “Yes, regarding drug-related intervention, trafficking in persons… (P54)”; “Yes, in the psychological field (P31)”.

Due to a lack of specification, the small variety (9.31%) response category was added, highlighting the need to expand the training offerings. Participants expressed, “There are usually no problems, but the training offered could be expanded” (E4). It also has a financial implication as almost every training incurs an economic cost: “It is difficult to find free and adapted to my situation” (P41); becoming a criterion for their selection or not: “only depends on the economic cost of the course” (P48). Finally, with less incidence to point out the access: “Maybe it should be given more visibility” (P45); the conciliation: “They are expensive and difficult to combine with the work” (P24); or the certificate: “when it is affordable comes from poorly accredited companies” (P40).

### 3.5 Modes of access to social educator's continuing training

The continued training offer may come from different agents or entities, and even in a self-taught manner, as can be seen in the data collected from the interview with social educators in practice in Table 5. According to this data, self-education prevails, followed by the offer of vocational school, and the university is little regarded as a source of continuing education.

**Table 5:** Modes of access to social educator's continuing training.

**Category**	***N***	**%**	**Category**	***N***	**%**
Own search	25	33.33%	Company	6	7.98%
Professional school	10	13.33%	University	3	3.99%
Foundation	8	10.64%	Tripartite	1	1.33%
Public Offer	6	7.98%			

Starting from a more detailed view of the data and focusing on the narratives of the social educators, it is worth noting that 33.3% of the respondents claim to be self-taught and carry out their own search for information to update themselves on certain professional topics. Several of the participants highlighted that the independent search for qualification processes taking into account the subject, labor requirements, and the cost: “Seeking information continuously about the problems associated with the area of my work” (P6); “I look for all kinds of courses on my own, without excessive price” (P36); “I find any kind of training when I need it” (P65). Similarly, the importance of the Internet as a space for the promotion of independent search is also noted: “I use training offers that I can find on the Internet. It satisfies them correctly (P35)”.

In addition to their own training, social education professionals also use the training plan offered by the professional school in each autonomous community (13.33%). The way in which the respondents refer to this category has been as follows: “Those of the professional college of the Valencian community” (P19); “I can carry out the training that is proposed from the inclusion service in the Regional Training Centre to the Faculty of Castile La Mancha. Also, INTEF courses” (P57).

Participants expressed that foundations and organizations (10.64%) where social educators work are an excellent training platform for the professionalization of social educators: “We have an offer that the Foundation offers us that covers many topics and adapt to our needs in general lines” (P4). These institutions also invest in the continued training of their workforce: “Red Cross and my host project pay much attention to continued formation” (P49), and pay special attention to the needs related to the collective that they work: “The Foundation favors continuous training in topics related to that with which they work” (P8).

### 3.6 Themes for continuing education according to the social educator

Table 6 shows the topics that social educators indicate as the most important to be addressed in their continuing education. Specifically, a total of 14 categories were compiled. Health and management was the most requested, followed by professionalization and dissemination. The dimensions of the type of intervention and the dimensions of the person stand out as the most chosen, much more than the contexts of intervention dimension.

**Table 6:** Key themes to be addressed in the continuing training of social education professionals.

**Dimension**	**Category**	***N***	***%***
Type of intervention (*N* = 64; 39.02%)	Management	23	14.02%
	Communication	14	8.53%
	Professionalization	14	8.53%
	Mediation	11	6.70%
	Intervention processes	2	1.21%
Contexts of intervention (*N* = 35; 21.34%)	Centers for minors	13	7.92%
	Dependency centers	12	7.30%
	Women's centers	8	4.87%
	Immigration centers	2	1.21%
Person dimensions (*N* = 65; 39.63%)	Health	37	22.56%
	Leisure and free time	9	5.48%
	Social relations	6	3.65%
	Family	6	3.65%
	Work	6	3.65%

Health is one of the topics most demanded by social educators as desired training (22.56%), focused mainly on mental health to address mental disorders (P13), to provide better support (P43), or simply to learn about the different problems and their solutions related to mental health (P23; P39). Leisure (5.48%) is also important, and it is highlighted in topics such as art (P21), sports (P65), or new technologies (P55). Social educators mentioned (3.65%) relationships, family, and work as other topics on which they currently need training.

Regarding the dimension of a practical nature, the type of intervention, management (14.02%) through legislation and procedures, is the most mentioned by the participants. They mention the following premises as justification for this subject in the future: knowledge of specific laws (P19), management, assessment and presentation of projects (P34), institutional networking (P40), or knowledge of legislative terminology (P56). Communication also received prominence in the training needs of the social educator (8.53%), in particular, delimiting different facets of language ranging from verbal (P48; P58) to non-verbal (P4; P21). The professionalization (8.53%) of the social educator (18.62%) is another of the most selected areas as a theme in this dimension. This stems from the need to forge a social educator who is a community agent (P25), develop a complete and more multidimensional role (P36), or develop resilience as a tool for better performance in their profession (P45). Finally, mediation (6.70%) and intervention guidelines (1.21%) were mentioned as other topics in demand, although with much less importance than the rest.

Centers for minors (7.92%) stand out in the dimension of context of intervention as the most selected category as interviewees expressed the need to develop better guardianship of minors in the following issues: educational performance, prevention of health disorders, improvement in relationships, labor orientation or related legal procedures (E15; P37; P39; P17). Centers with drug dependence (7.30%) are another of the contexts that were highlighted. Social educators wanted to know what specific programs there are (P37), the profiles and substances (P42), and learn prevention technologies (P15). Women's centers (4.87%) and immigration centers (1.21%) are the contexts that were least considered as future training topics.

## 4 Discussion and conclusions

The relationship between training and professionalization is complex, although they are closely connected; hence, they are discussed by various studies (Solís-Galán and López-Andrada, 2020). The current study presented the perception of social education practitioners regarding their continuing training needs, as well as the aspects of improvement and the thematic lines on which it should focus.

In relation to the first of the proposed objectives, the professional in social education recognizes the need and usefulness of the initial training in professional performance, as well as considering that updating training is a way of improving their professional practice. Specifically, despite the role of the social educator being recognized in previous studies to include establishing intercultural bridges and promoting the participation of immigrants and other educational agents in various socio-educational environments to strengthen coexistence and integration (Bretones et al., 2014), the data obtained show the need to emphasize and deepen aspects related to the types and contexts of social education in university studies on social education. Interventions prioritizing older adults, minors, and women over the immigrant group are important. The peculiarities of socio-educational intervention with highly vulnerable groups vindicate university students with a strong vocation, but this does not always happen this way. Hence, it is important to incorporate immersive practices in work contexts that allow screening among future social educators, avoiding future frustration once the degree is finished. Without a vocation in the student of social education, the degree can be unsustainable, increasing the rates of university school failure. However, other aspects, such as the economic situation, state of health, and participation in university life, also affect the dropout rate in this discipline (Portal Martínez et al., 2022).

Another recurrent turning point in the initial training of the social educator focuses on the debate between theory and practice. The qualitative data obtained in this study show a significant approach to practical content within the framework of the degree, claiming a greater number of credits that allow more time to be able to learn about various work areas experientially, coinciding with what was stated by Díaz-Puppato (2019). Among the uses of this practical training, practicing social educators point out that it provides tools, technologies, and strategies for direct intervention. However, and in contrast to what was made explicit by the professionals in this study, it is evident in the interview carried out with those responsible for curricular design in the university degree in social education that there is a lack and the need for a greater foundation and consultation of the bibliographic sources that support the materials used in the training. This absence of bibliographic sources further evidences the tensions between theory and practice (Díaz-Puppato, 2019).

Therefore, it can be generally concluded that social education practitioners believe that continuing training is a valuable tool to consciously improve their professional skills, which strengthens and reinforces their commitment to their daily work. The social education practitioner is also able, according to the results of the present study, to highlight themes vs. skills, which not only points to shortcomings (the ones of the demanding themes that they formulate) but also allows to identify the strengths (in what they do not contemplate in their demands) of training systems and personal efforts that have allowed them to move forward and face the situations in which they must intervene professionally.

The variety in the categorization of answers in this study is valuable in itself: different people incorporate different experiences and demands according to the realities in their lives. Nevertheless, certain “fashioned” topics persist that require further study for proper analysis because their extended offer contributes, without doubt, to being part of what is commonly considered in the training paths, without necessarily being an important key for improving the specific professional practice.

In detail, and taking into account the specific objectives formulated, the analysis that the testimonies provide of the initial training received in the Grade of Social Education proves, as a first conclusion, the justification and necessity of continuous training in a constantly changing world. Various studies have demonstrated this need previously (Martínez-Pérez et al., 2024). It has been reaffirmed by 90% of the professionals involved in this work, recognizing that the initial training received requires some improvement and potentially can be realized either in sectors or in interventions, which coincides with the conclusions found in recent studies (Eslava-Suanes et al., 2018a).

The high percentage of professionals who indicate that there is an initial training that can be improved does not imply excessive unanimity on the objective of such improvement. The percentages are quite dispersed; only one topic reached a representation of close to 15%. The variety of responses is large, so it cannot be said that there is a clear characterization of the alleged shortcomings of the offer received. Of the 19 categories found, 12 remain with representations of <10% of the sample. This diversity and lack of consensus is not surprising when we view the social educator as a highly versatile professional to address discrimination in all its forms, with a marked pedagogical character that involves the creation of educational environments and the implementation of mediating and training actions (Pinto Dos Santos, 2023). At the same time, the demand for more training in the degree on the collectives to be addressed deduces an intention to think of the educator or educator based on profiles and not as the professional able to address problem situations around subjects independent of the collective to which he belongs. All of this also allows us to refer to the initial considerations about the imperative need for systemic contextualization (Escudero Muñoz, 2020). This involves observing the daily reality of the social educators who are devoted to addressing problematic situations for which there is not enough institutionalized or specialized intervention, particularly in areas such as mental health, which simply does not correspond to their roles. The shortcomings or gaps in this offer of assistance adjusted to social realities very often become the battle horse of the social educator in practice, as some studies (González et al., 2016) suggest. Although training can mitigate the discomfort that this situation generates, the optimization of the response happens because the administrations listen to the account of the professionals in practice, value a diagnosis that establishes the reality of the existing problems supported in increasing specific cases, and bet on taking responsibility (political, economic, social, institutional, and professional) of the situations that require urgent responses.

The second objective focused on knowing the continuing and permanent training needs identified at the beginning of the professional career. Prior training decisively influences the trajectory of the continuing education of the educator and social educator, in addition to the moment and context in which they develop. Analyzing the various previous studies on this subject, particularly the studies of Sáez (2004) and Haba (2021), it is observed that the training needs of social educators predominantly focused on understanding work, problem-solving, and collaboration with other professionals. However, 20 years later, these needs were more related to specific aspects of the professional sphere. In the current study, the perception has not varied significantly, though linked to new target groups such as minors and areas such as mental health and drug addiction. This raises questions that compel reflection on the profession of social education: Do we really focus on the functions and competencies of the educator and the social educator when we think about this profession? Or do we confuse the need to raise specific learning goals, depending on the problem situation, with the specialization or dissection of the profession? Do we detect the needs of the teacher or social educators or those derived from assuming functions that do not correspond? Therefore, coordination is needed at the level of initial continuing training and professional performance.

Knowledge about legislation was indicated as the main training gap in this study. This finding reinforces those of other studies, such as that of Eslava-Suanes et al. (2018a), that, through focus groups, have identified similar training gaps in practicing social education professionals. Mediation was identified as one of the training needs at the beginning of professional practice. This need corroborates the data from studies that, for school settings, insist on the significantly greater presence of social educators in conflict mediation and coordination of activities that favor the relationship of the members of the educational communities (González et al., 2016).

The third objective of this study related to participation in lifelong learning experiences. The results obtained do not facilitate an evaluation. The needs identified in the previous category of this study are directly related to the type of training topics the surveyed social education professionals have accessed. It is noticeable that “mediation,” which is one of the specific functions of the educator and social educator, and for which there is supposed to be training from the Graduate level, is detected as a continuous training need. Our findings also highlight the need for training related to “gender and sexual education,” responding to the times that we are living in. Further, specifically focusing on minors and topics such as mental health and drug addiction, it is important to address the question that can lead us to reflect on the professionalization of educators and social educators: Is an educator or social educator necessary for each topic? From our point of view, the affirmative answer to that question results in a dissection of the profession that has nothing to do with it, and that loses meaning from a professional logic. In some previous field studies, even the professionals recognized that this training offer is governed by a trend or fashion (Eslava-Suanes et al., 2018a).

Once again, taking advantage of the results concerning identifying difficulties in seeking training courses focused on social education (objective four), it would be necessary to ask whether it is specific training needs or the search for answers to learning goals raised from a specific situation. Specific training for specific groups is again being demanded, giving rise to thinking about the labor jurisdiction of social education and the delimitation of functions between the different socio-educational professions. The fundamental difficulty pointed out in this study, relating to identifying training that meets specific needs and does not take refuge in theory but in an approach that connects theory and practice, coincides with other contributions of inquiry through interviews in field studies of social education professionals (Eslava-Suanes et al., 2018a).

The findings from this study, particularly about continuing training, echo the words of Escudero Muñoz (2020, p. 113): “Either it is raised and committed as a right and a duty, as a personal, social and institutional task, or it will continue to depend on particular choices and decisions, occasional and voluntary, capable of undermining joint projects.” This thesis is reinforced if we consider that two of the variables that prevent social education professionals from becoming mere workers in institutions are training and autonomy. Some authors argue that the limitations in this regard derive from the de-professionalization and even proletarization of social education professionals.

The reality of the agents involved in the continuing training of social educators in Spain shows specific characteristics that are quite distant from other practices in relatively close contexts (Eslava-Suanes et al., 2018b). In relation to the available training offer addressed to social educators and the degree of satisfaction (fifth objective), the social educator's own initiative in the search for continued training is highlighted, which refers to a commitment to their own professional updating. It also highlights the positive evaluation of the initiatives of some of the organizations where social educators work, with their own training plans adapted to the needs of their functions. This call to the need for specific plans that can be integrated into proposals that allow for the improvement of the work centers themselves in the framework of the design of training itineraries (Tejada Fernández, 2018) that respond less to the immediate and more to the consistency of a training need in time, are still today outstanding challenges in our field.

Finally, regarding the identification of key themes in the continuing training of social education practitioners (sixth objective), the results show that social or systemic factors have permeated the narratives of social educators about their continuing education. Thus, the socially perceived and denounced “evils” in different professionalizing areas, such as the excessive and increasing bureaucratization of our Western administrative systems, are present in their testimonies significantly (Precht, 2022).

In this regard, one of the topics most considered in scientific production as necessary to be worked competently in continuing training is recycling in ICTs, as evidenced by Cabezas et al. (2020), Gómez-Galán et al. (2020), and Martínez-Pérez et al. (2024) among others. As a professional who assumes the responsibility of mitigating the digital divide and social exclusion, especially in risky environments, the social educator requires practical training (Martínez-Pérez et al., 2024). They are responsible for promoting digital competence to facilitate the adaptation of people to contemporary society, and their attitude toward them is conditioned by professional variables rather than by personal variables such as age and gender (Cabezas et al., 2020). However, despite mentioning ICT among the topics, the practicing social educators involved in this study do not place it in their testimonies among the preferred or priority ones.

Finally, in the results obtained, a strong tendency is observed to situate the training of the social educator, both initial and continuous, linked to the areas of intervention, leaving aside the educational situations of the school. However, there is a strong trend in previous literature in the professional field of social educators that has long contemplated the great challenge of incorporating this in educational centers (Machalík, 2020; Díez-Gutiérrez and Muñiz-Cortijo, 2022). All this is supported by the current educational crisis, mainly due to the resistance and adaptation of schools to the characteristics and needs of postmodern societies and their role as agents of change, mediating between students, families, teachers, and the social environment, promoting democratic values and preparing for integration and coexistence in society (Gallardo-López and López-Noguero, 2020). This shows the need for field studies in the professional practice of the social educator to identify new sources of employability.

The over-saturation of time favors the collection of information, damaging even more if it is for research of a qualitative nature. In this sense, the collection of oral information provides a greater depth of narratives than those that require written expression. Sometimes, qualitative research becomes the forefront of quantitative research (Bolívar Botía, 2002). In this way, the data obtained can be useful for designing quantitative instruments that deepen the training needs of professionals in social education. The very reality of the social educator is so complex that it sometimes leads him to perform different roles in the workplace (educational, re-educative, informative, guidance, animation, management, local development, project design, intervention, mediation, etc.), to respond to emerging needs (Pinto Dos Santos, 2023), to respond, ultimately, to situations, in a professional exercise as exciting as complex.

## Data availability statement

The data analyzed in this study is subject to the following licenses/restrictions: personal information. Requests to access these datasets should be directed to josesantiago.alvarez@um.es.

## Ethics statement

The studies involving humans were approved by Comité Ético Universidad de Murcia. The studies were conducted in accordance with the local legislation and institutional requirements. The participants provided their written informed consent to participate in this study.

## Author contributions

MC: Conceptualization, Funding acquisition, Project administration, Resources, Validation, Visualization, Writing – original draft. AA: Conceptualization, Funding acquisition, Investigation, Project administration, Resources, Supervision, Validation, Visualization, Writing – original draft. JÁ: Conceptualization, Data curation, Formal analysis, Funding acquisition, Investigation, Methodology, Resources, Software, Supervision, Validation, Visualization, Writing – original draft, Writing – review & editing. MªH: Conceptualization, Data curation, Formal analysis, Funding acquisition, Investigation, Methodology, Resources, Software, Supervision, Validation, Visualization, Writing – original draft, Writing – review & editing.
